# Swine coronaviruses (SCoVs) and their emerging threats to swine population, inter-species transmission, exploring the susceptibility of pigs for SARS-CoV-2 and zoonotic concerns

**DOI:** 10.1080/01652176.2022.2079756

**Published:** 2022-06-19

**Authors:** Jigarji C. Thakor, Murali Dinesh, Rajendran Manikandan, Suresh Bindu, Monalisa Sahoo, Diptimayee Sahoo, Manish Dhawan, Megha Katare Pandey, Ruchi Tiwari, Talha Bin Emran, Kuldeep Dhama, Wanpen Chaicumpa

**Affiliations:** a Division of Pathology, ICAR-Indian Veterinary Research Institute, Bareilly, Uttar Pradesh, India; b Immunology Section, ICAR-Indian Veterinary Research Institute, Bareilly, Uttar Pradesh, India; c Department of Microbiology, Punjab Agricultural University, Ludhiana, India; d The Trafford Group of Colleges, Manchester, United Kingdom; e Department of Translational Medicine Center, All India Institute of Medical Sciences, Bhopal, Madhya Pradesh, India; f Department of Veterinary Microbiology and Immunology, College of Veterinary Sciences, Uttar Pradesh Pandit Deen Dayal Upadhyaya Pashu Chikitsa Vigyan Vishwavidyalaya Evam Go Anusandhan Sansthan (DUVASU), Mathura, India; g Department of Pharmacy, BGC Trust University Bangladesh, Chittagong, Bangladesh; h Center of Research Excellence on Therapeutic Proteins and Antibody Engineering, Department of Parasitology, Faculty of Medicine Siriraj Hospital, Mahidol University, Bangkok, Thailand

**Keywords:** Pig, porcine, Coronaviruses, SARS-CoV-2, zoonosis

## Abstract

Swine coronaviruses (SCoVs) are one of the most devastating pathogens affecting the livelihoods of farmers and swine industry across the world. These include transmissible gastroenteritis virus (TGEV), porcine epidemic diarrhea virus (PEDV), porcine respiratory coronavirus (PRCV), porcine hemagglutinating encephalomyelitis virus (PHEV), swine acute diarrhea syndrome coronavirus (SADS-CoV), and porcine delta coronavirus (PDCoV). Coronaviruses infect a wide variety of animal species and humans because these are having single stranded-RNA that accounts for high mutation rates and thus could break the species barrier. The gastrointestinal, cardiovascular, and nervous systems are the primary organ systems affected by SCoVs. Infection is very common in piglets compared to adult swine causing high mortality in the former. Bat is implicated to be the origin of all CoVs affecting animals and humans. Since pig is the only domestic animal in which CoVs cause a wide range of diseases; new coronaviruses with high zoonotic potential could likely emerge in the future as observed in the past. The recently emerged severe acute respiratory syndrome coronavirus virus-2 (SARS-CoV-2), causing COVID-19 pandemic in humans, has been implicated to have animal origin, also reported from few animal species, though its zoonotic concerns are still under investigation. This review discusses SCoVs and their epidemiology, virology, evolution, pathology, wildlife reservoirs, interspecies transmission, spill-over events and highlighting their emerging threats to swine population. The role of pigs amid ongoing SARS-CoV-2 pandemic will also be discussed. A thorough investigation should be conducted to rule out zoonotic potential of SCoVs and to design appropriate strategies for their prevention and control.

## 1. Introduction

The transmission of zoonotic diseases has become the world’s most serious threat to public health. As pathogens of importance on a global level, zoonotic CoVs have a higher tendency for cross-species transmission to humans and other important domesticated species (Edwards et al. 2020). Coronaviruses (CoVs) cause a variety of diseases in both animals and humans, and their ability to cause dreadful diseases in livestock and companion animals prompted extensive research in the twentieth century. Coronaviruses are a wide group of mammalian and avian pathogens identified in 1968 (Almeida et al. 1968; Alluwaimi et al. 2020). They belong to the order *Nidovirales, Coronaviridae* family, and *Coronavirinae* subfamily (Alluwaimi et al. 2020). Since CoVs infect a broad range of habitats, several species of animals carry these pathogens as well as affects humans; causing respiratory, enteric, or systemic diseases in a variety of mammalian hosts that vary in clinical severity from subclinical, mild, moderate to fatal (Cui et al. 2019; Andersen et al. 2020; Dhama et al. 2020; Ghosh and Malik 2020; Haake et al. 2020; Colina et al. 2021; Zhang et al. 2021a). Coronaviruses have been reported to cause infection in diverse species of animals including cattle, swine, horses, cats, dogs, camels, rabbits, rodents, bats, palm civets, ferrets, mink, snake, avian/poultry species as well as wildlife (Kahn and McIntosh 2005; Dhama et al. 2014a, 2014b; Fehr and Perlman 2015; Miłek and Blicharz-Domańska 2018; Haake et al. 2020; Malik et al. 2020; Colina et al. 2021; Zhang et al. 2021a). During the past two decades, three deadly and most pathogenic CoVs have affected humans namely, severe acute respiratory syndrome coronavirus (SARS-CoV) (2003), Middle East respiratory syndrome coronavirus (MERS-CoV) (2012), and severe acute respiratory syndrome coronavirus 2 (SARS-CoV-2) (2019), the last one as the cause of ongoing coronavirus disease 2019 (COVID-19) pandemic (Ksiazek et al. 2003; Berger et al. 2004; Dhama et al. 2020; Rabaan et al. 2020, 2021; Shaw et al. 2020).

**Figure 1.**
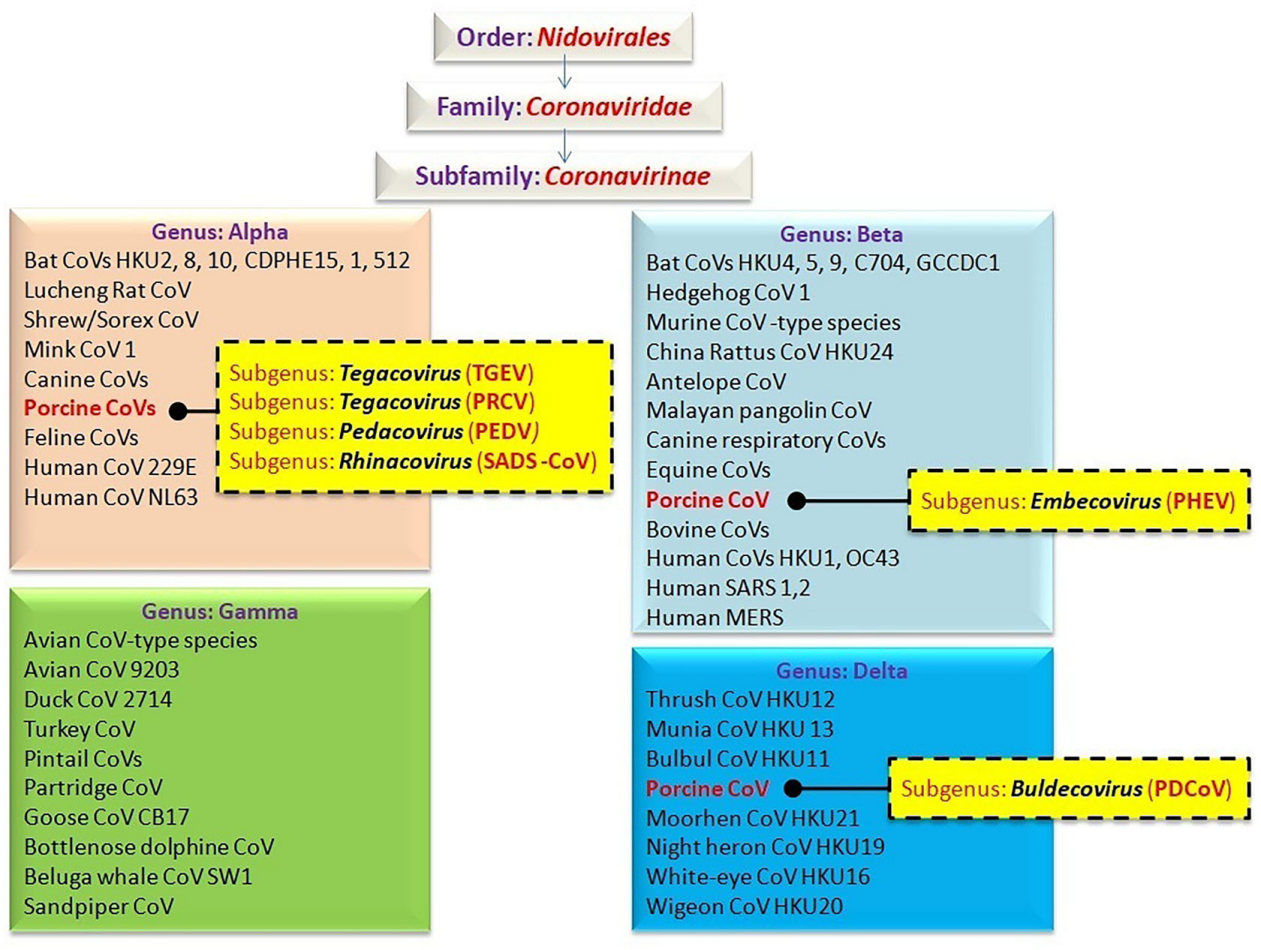
Systematic classification of important coronaviruses affecting animals and humans.

Swine CoVs (SCoVs) are the major enteric and respiratory pathogens in swine. Nevertheless, cardiovascular, gastrointestinal, as well as peripheral and/or central nervous system illnesses are also noticeable in the vast majority of instances. Four CoV genera have been identified based on genomic criteria: *Alphacoronavirus*, *Betacoronavirus*, *Gammacoronavirus*, and *Deltacoronavirus* (Chen et al. 2020). Six swine CoVs have been reported among them: (1) the transmissible gastroenteritis virus (TGEV), (subgenus *Tegacovirus*) which was first identified in 1946; (2) the porcine hemagglutinating encephalomyelitis virus (PHEV) (subgenus *Embecovirus*) identified in 1957; (3) the porcine epidemic diarrhea virus (PEDV) (subgenus *Pedacovirus*), found in 1977; (4) the porcine respiratory coronavirus (PRCV), a variant of TGEV discovered in 1984; (5) the porcine delta coronavirus (PDCoV) (subgenus *Buldecovirus*) reported in 2012; and (6) swine acute diarrhea syndrome coronavirus (SADS-CoV) (subgenus *Rhinacovirus*), discovered in 2017 (Decaro et al. 2020). Detailed and systematic classification of CoVs is depicted in Figure 1. Four of the Swine coronavirus, i.e. TGEV, PRCV, PEDV, SADS-CoV belong to genus *Alphacoronavirus,* PHEV to genus *Betacoronavirus* while PDCoV to genus *Deltacoronavirus.* Concomitant infection with TGEV, PDCoV, PEDV and SADS-CoV are common in infected pig farms resulting in acute gastroenteritis (Luo et al. 2020). However, PRCV can also cause pneumonia (Boley et al. 2020). The following factors contribute to increased susceptibility of neonatal pigs to CoV infection namely: the immune system of a young pig is not completely developed; the stomach pH of younger pigs is less acidic than that of older pigs; the regeneration of enterocytes that line the intestinal villi through progenitor cells is slower in younger pigs; piglets are more vulnerable to fluid and electrolyte imbalances due to mal-digestion and intense malabsorption (Doyle and Hutchings 1946; Turlewicz-Podbielska and Pomorska-Mól 2021). Porcine epidemic diarrhea virus is considered to be more pathogenic than PDCoV, and the mortality rates of PEDV and PDCoV in newborn piglets are about 80–100% and 40%, respectively (Jung et al. 2016; Niederwerder and Hesse 2018). In China, SADS-CoV mortality was high (90–100%) in piglets younger than 5 days old, but it was reduced to 5% in piglets older than 8 days (Zhou et al. 2018; Xu et al. 2019).

**Figure 2.**
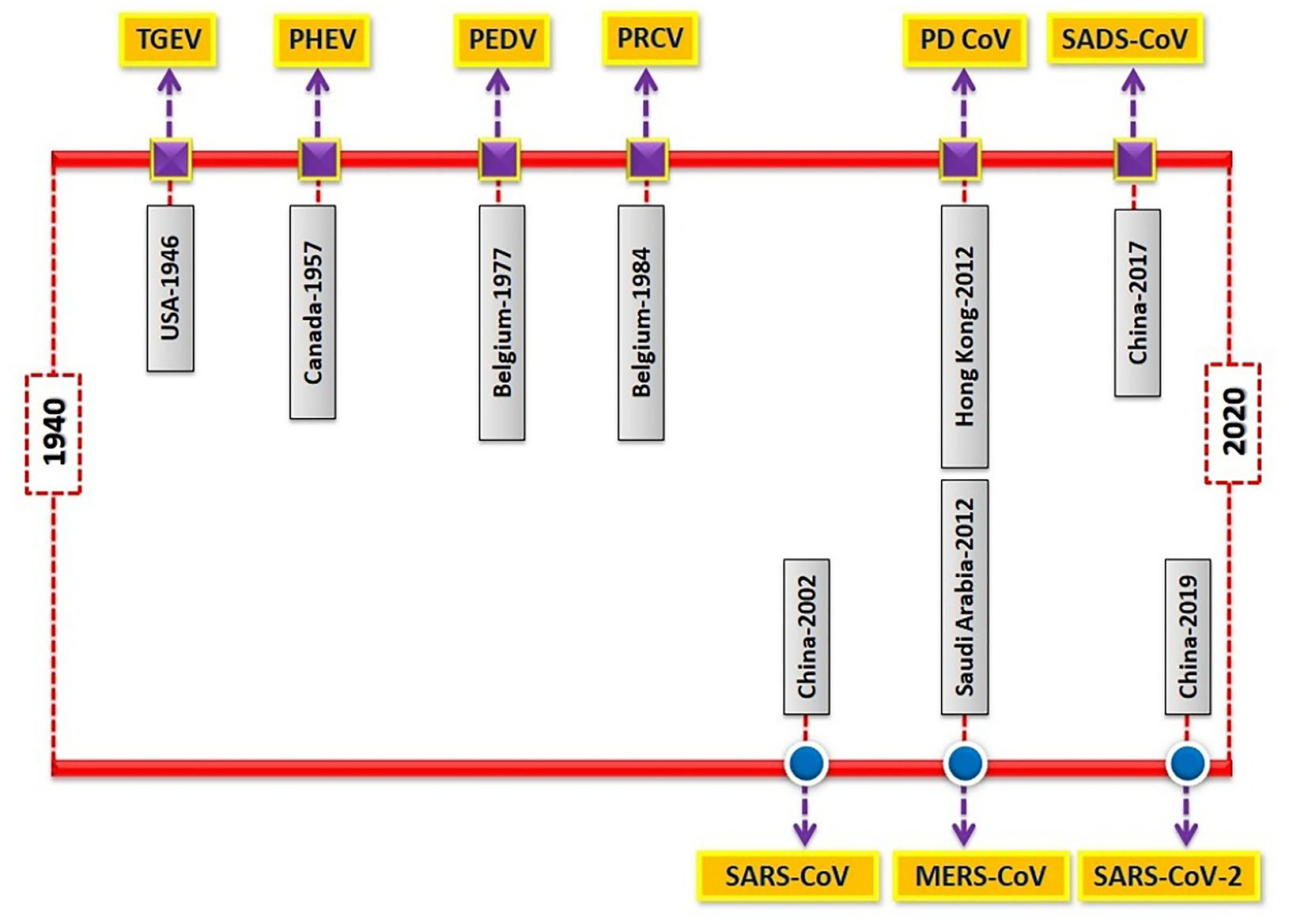
The chronological time-line of events presenting major coronaviruses outbreaks in swine (upper bar) and humans (lower bar).

Outbreaks of SCoVs are more common in winter and spring seasons. Piglets are more susceptible to infection. The disease is characterized by severe clinical symptoms such as diarrhea, vomiting, anorexia, dehydration, metabolic acidosis, hyperkalemia and further it may lead to death. Villus destruction and multifocal necrosis of intestinal epithelium are the most noticeable pathological features of SCoV infection (Luo et al. 2017). Coronaviruses occur as quasispecies with a high degree of genetic recombination and mutation (Forni et al. 2017). This promotes the introduction of newer strains with changes in cell tropism and host specificity. Virus spreading from wild animal sources is a significant threat to human and animal health (Olival et al. 2017). The majority of SCoVs are transmitted by fecal-oral route, whereas PRCV is transmitted primarily through inhalation (Saade et al. 2020). Bats have been identified as the primary reservoirs for emerging coronaviruses (Zhou et al. 2020a; Frutos et al. 2021). In 2003, SARS-CoV pathogen emerged in bats and then transmitted to humans via intermediate host (civet cats) (Peiris et al. 2003). The direct interaction between animals and humans paves the way for adaptive mutation and interspecies transmission (Lu et al. 2015). Chronological reports of major CoVs outbreaks in humans and pigs have been illustrated in Figure 2.

Coronaviruses have posed devastating consequences on commercial animal production over the past few years, including infectious bronchitis virus (IBV) in poultry, bovine CoV as well as the emergence and re-emergence of multiple CoVs in swine such as TGEV, PEDV, and PDCoV. These animal CoVs infections facilitate understanding CoV disease as a whole; most of the animal CoVs have complex pathogenesis alike SARS-CoV-2 and thus can also throw light on the SARS-CoV-2 ongoing outbreaks amid COVID-19 pandemic and zoonotic concerns (Kenney et al. 2021). This review article presents an introductory overview on coronaviruses with a particular focus on swine coronaviruses (SCoVs) and their prevalence and epidemiology, virology, replication, evolution and emergence, clinical signs, pathology and pathogenesis, interspecies transmission, wildlife reservoirs, spill-over events and feasibility of the role of pigs in SARS-CoV-2 transmission during the ongoing pandemnic, and highlighting an overall scenario of emerging threats of SCoVs to swine population and human health.

## 2. Occurrence and epidemiology

Over decades, PRCV, TGEV and PHEV are prevalent in swine. While PDCoV, PEDV and SADS-CoV are emerging or re-emerging these CoVs pose a serious threat to pig farming. Chimeric PEDV and TGEV strains have also been documented in Italy, Germany, Slovakia, and Spain (Akimkin et al. 2016; Boniotti et al. 2016; Valko et al. 2019; de Nova et al. 2020). PEDV outbreaks were reported in Mexico, Czech Republic, Hungary, Belgium, China, Korea, Italy and Thailand during 1990s. It re-emerged as a major swine disease in China (Theuns et al. 2015; Niederwerder and Hesse 2018; Reveles-Felix et al. 2020). According to the most recent prevalence study in China, the average PEDV-positive rate in pigs from 39 farms across eight provinces, primarily in Hunan, was 38.0% (Tan et al. 2020). Later on, PEDV mutants were reported in Asian countries (Thailand, South Korea, Vietnam, and the Philippines) (Lee and Lee 2014; Cheun-Arom et al. 2016; Diep et al. 2018; Garcia et al. 2018), North America (Canada, Mexico) (Ojkic et al. 2015; Trujillo-Ortega et al. 2016) and in European countries (Austria, Ukraine, Belgium, Germany) (Steinrigl et al. 2015; Pensaert and Martelli 2016). Transmissible gastroenteritis virus and PEDV were co-circulating in Eurasia and the United States (Akimkin et al. 2016; Belsham et al. 2016; Mandelik et al. 2018). A pathogenic recombinant PEDV/TGEV mutant (swine enteric coronavirus, SeCoV) was found in Europe (Belsham et al. 2016; Boniotti et al. 2016). Even though, TGEV epidemiological studies are lacking in many countries (Valko et al. 2019). PRCV which is a TGEV mutation with a naturally occurring S gene deletion (170 to 190 kDa) has been reported in many European countries, including the Netherlands, Denmark, the United Kingdom, France and Spain (Magtoto et al. 2019; Turlewicz-Podbielska and Pomorska-Mól 2021). Porcine hemagglutinating encephalomyelitis virus, the only swine *Betacoronavirus*, is prevalent worldwide (Dong et al. 2014; Lorbach et al. 2017; Li et al. 2016). However, as per study in the United States of America, unusual respiratory (influenza-like) symptoms and the increasing prevalence of PHEV in adult swine could indicate a possible shift in their tropism, which could lead to a significant change in its epidemiology (Lorbach et al. 2017). Clinical outbreaks of vomiting and wasting disease (VWD) or PHEV-related fatalities were first reported in Canada in 1957 (Roe and Alexander 1958; Mora-Diaz et al. 2019). Later, clinical cases were recorded in many countries, including Argentina, the United States of America, Belgium, China, Japan, and South Korea (Gao et al. 2011; Dong et al. 2014; Mora-Diaz et al. 2019). During 2017 in Southern China, a unique bat-HKU2-like pig coronavirus (GDSO4 strain), i.e. porcine enteric *Alphacoronavirus* (PEAV), causing severe diarrhea in suckling piglets was reported (Gong et al. 2017). It was later known as swine acute diarrhea syndrome CoV (SADS-CoV) (Zhou et al. 2018).

Infection with PDCoV has been documented in several countries including the United States of America (Li et al. 2014; Wang et al. 2014; Ma et al. 2015), Canada, South Korea (Lee and Lee 2014; Lee et al. 2016), China (Liu et al. 2017; Li et al. 2018a; Zhang et al. 2019), Thailand (Madapong et al. 2016; Lorsirigool et al. 2017; Saeng-Chuto et al. 2017), Lao People’s Democratic Republic (Lorsirigool et al. 2016), Vietnam (Le et al. 2018), Mexico (Perez-Rivera et al. 2019), and Korea (Lee et al. 2016) causing serious threat to pig industry. In diarrheic pigs, the prevalence of PDCoV in China and the United States of America has been reported to be as high as 30 and 7%, respectively; and coinfections with PEDV are common in about 60% of cases (Zhang et al. 2019; Turlewicz-Podbielska and Pomorska-Mól 2021). Between 2015 and 2017, a study showed a prevalence rate of 36.2% of PDCoV in fecal samples from nine Chinese provinces (Ding et al. 2020). In Henan Province, China, researchers conducted a passive surveillance study to determine the evolutionary relationship between PDCoV strains. Phylogenetic analysis of the complete genome, nucleocapsid, and spike gene sequences showed that Chinese clade Henan strains of PDCoV were more closely related to other reference strains of PDCoV (Zhang et al. 2019).

Transmissible gastroenteritis virus (TGEV) was first reported in Belgium in 1984 and was first detected in the United States of America in 1946 (Doyle and Hutchings 1946; Pensaert et al. 1986). Cases of acute diarrhea in piglets in the United States of America led to the identification of TGEV in 1946 (Doyle and Hutchings 1946), and was later reported in Asia, Africa, South America and Europe resulting in significant economic losses in pig breeding sector (Kim et al. 2000; Stevenson et al. 2013; Cheng et al. 2020). Even though vaccines are widely used, TGEV infections are the primary source of enteric infection and mortality in piglets in the United States of America and around the globe from the 1960s to the 1980s (Yaeger et al. 2002). The existence and widespread occurrence of PRCV (deletion variant of TGEV), limited the therapeutic effect of TGEV (Turlewicz-Podbielska and Pomorska-Mól 2021). The enteropathogenic TGEV causes extreme diarrhea that is limited in swine. Even though pigs of every age group are prone to diseases, piglets exhibit the most severe clinical symptoms, with an almost 100% mortality rate. Due to development of resistance, the mortality rate decreases with age (Moon 1978; Turlewicz-Podbielska and Pomorska-Mól 2021). Piglets that survive from TGEV infection can recover within six to eight days, but virus can persist in lungs or gut upto 104 days and aid in the spreading of virus to non-infected pigs (Lawhorn 1999; Xia et al. 2018).

Unlike TGEV, PRCV has a preference for the respiratory system. A survey conducted in Belgium in 1984 led to the discovery of this virus. In the absence of vaccination, there was an increase in the number of animals with TGEV antibodies (up to 68%). Three years later, the virus had infected 100% of Belgium's swine farms (Enjuanes and Van der Zeijst 1995). The PRCV has been observed in a number of European countries, including the Netherlands, Denmark, the United Kingdom, Spain, and France (Enjuanes and Van der Zeijst 1995). Serological tests detected PRCV for the first time in Indiana, USA in 1989 (Enjuanes and Van der Zeijst 1995). Other PRCV isolates have since been reported in the USA and Canada (Enjuanes and Van der Zeijst 1995; Turlewicz-Podbielska and Pomorska-Mól 2021).

Porcine epidemic diarrhea virus (PEDV) was defined in island located at United Kingdom in 1977 (Niederwerder and Hesse 2018). In 1978, a ‘Coronavirus-like’ agent distinct from other identified SCoVs was reported (Chasey and Cartwright 1978). PEDV origin is unclear. There is no conclusive proof that bats introduced it into the swine farm in the 1970s. PEDV can replicate in cell lines from pigs, monkeys, bats, ducks, and humans, indicating that the virus has breached the interspecies boundary amongst bats and pigs (Liu et al. 2015; Teeravechyan et al. 2016). Porcine epidemic diarrhea virus was discovered in feral hogs in the Korean peninsula and the USA (Bevins et al. 2018). The virus had spread to most swine-producing areas within a year of its emergence, killing nearly 7 million pigs, mostly neonates in their first few weeks of existence. Porcine epidemic diarrhea virus has caused significant economic losses; with estimates of $300,000 in annual losses for a single 700-sow farrow-to-finishing herd (Weng et al. 2016). Infected pigs are the major source of infection to others, but it can also be spread through contaminated water or food, automobiles, shoes, and other inanimate objects. Workers involving in farm activities can similarly serve as vehicles to transmit viruses to newborn pigs (Dee et al. 2014, 2016; Schumacher et al. 2016). There has been some proof of PEDV transmission via aerogenous route (Alonso et al. 2014; Jung et al. 2020) but no other studies have described it till now. Currently, three PEDV genotypes are identified on the basis of S gene: I) G1a PEDV, which includes mildly virulent strains from Europe and Asia; ii) G2 PEDV, also known as ‘Original USA PEDV’, which includes highly lethal strains which was disseminated from Asia, presently prevalent in the USA; and iii) G1b PEDV, which embody S-INDEL strains (strains with insertions and deletions in the S gene linked to mild clinical outbreaks) (Decaro and Lorusso 2020). Infectious virus excretion up to 14–16 days in four-week-old pigs affected with an evolving PEDV strain (non-S INDEL) (Crawford et al. 2015).

Porcine hemagglutinating encephalomyelitis virus (PHEV) was first found in the brain of a suckling piglet suffering from encephalomyelitis and cultivated in primary porcine kidney (PK) cells (Greig et al. 1962). Porcine hemagglutinating encephalomyelitis virus was derived from bovine CoV, which is thought to have descended from a bat virus via rodent adaptation (Decaro et al. 2020). Porcine hemagglutinating encephalomyelitis virus has been the only neurotropic virus known to affect pigs, and it was one of the first swine coronavirus to be isolated. Later, a virus antigenically related to PHEV was identified in Europe as Von Willebrand disease (VWD) virus. Porcine hemagglutinating encephalomyelitis virus and VWD virus were first SCoV identified as a cause of encephalomyelitis (Mora-Diaz et al. 2019). Porcine hemagglutinating encephalomyelitis virus was recently linked to an influenza-like respiratory disease from pigs in Michigan, USA according to a new study (Lorbach et al. 2017).

Swine acute diarrhea syndrome coronavirus (SADS-CoV) is an *Alphacoronavirus* that is most analogously related to coronavirus (HKU2) of bat, but is also related to HCoV NL63, HCoV 229E and PEDV (Gong et al. 2017). It was first identified and confirmed as the causal agents of a catastrophic pig epidemic in southern China in 2017, which killed nearly 24,500 piglets and resulted in significant commercial losses (Pan et al. 2017). Infection with SADS-CoV was linked to acute diarrhea and vomiting with mortality up to 90% in piglets under the age of five days (Zhou et al. 2019). Swine acute diarrhea syndrome coronavirus strain sequence resembles the HKU2 coronavirus of *Rhinolophus* spp. bats, implying that SADS-CoV may have originated from bats (Zhou et al. 2018).

Porcine *Deltacoronavirus* (PDCOV) is an emerging porcine enteric coronavirus which responsible for diarrhea in young pigs (Zhang et al. 2019) and reported in Hong Kong in 2012 (Woo et al. 2012). Porcine *Deltacoronavirus* has been shown to originate from sparrow CoV and known to be pathogenic to cattle and poultry apart from swine (Khamassi Khbou et al. 2021). Molecular clock analysis has shown that probable origin dated back to 1990s in South-east Asia with a common ancestry to sparrow CoV of around 1810 (Ye et al. 2020). But PDCoVs were previously identified in pigs and wild birds during preliminary investigations in China and Hong Kong from 2007 to 2011 (Boley et al. 2020). Yet, at the Chinese live-animal markets in 2005–2006 existence of PDCoV in small mammals like Asian leopard cats and Chinese ferret badgers were previously reported (Dong et al. 2007). Porcine *Deltacoronavirus* was found in diarrheic pigs on mainland China (Dong et al. 2015; Song et al. 2015). Interspecies transmission of PDCoVs is suspected amongst porcine, avian, and Asian carnivorous animals, because their S and helicase genes are closely linked to PDCoV (Li et al. 2018b). According to recent experimentation, PDCoV can cause infection and destroy cells from different animal and avian species (chicks and poults). Since avian susceptibility to PDCoV has been proven *in vivo*, but *in vitro* data indicating human susceptibility should be investigated further (Boley et al. 2020). The other swine enteric viruses including PEDV, TGEV and PDCoV infections cause vomition, diarrhea, dehydration and death in newborn piglets (Zhang 2016). In Ohio, USA the first epidemic of PDCoV-related diarrhea in pigs was recorded in 2014. Reverse trancription polymerase chain reaction (RT-PCR) testing revealed that 92.9% of diarrheic pigs intestinal or fecal samples from five Ohio farms were tested positive for PDCoV (Wang et al. 2014). In 2012, the first PDCoV strain was detected in Hong Kong during a CoV molecular epidemiology study that used RT-PCR to analyze pig samples. The complete genome sequencing revealed that the 2012 PDCoV strain sequences were very similar to the two Chinese prototype PDCoV strains (HKU15-155, HKU15-44) (Turlewicz-Podbielska and Pomorska-Mól 2021). Over the same time, two genetically related PDCoV strains (USA/IA/2014/8734 and SDCV/USA/Illinois121/2014) were identified in the USA (Li et al. 2014; Marthaler et al. 2014). Although this PDCoV has now been confirmed in 19 states of the USA (www.aphis.usda.gov/animal-health/secd); it is far less common than PEDV.

## 3. Genome and structure of swine coronaviruses (SCoVs)

**Figure 3.**
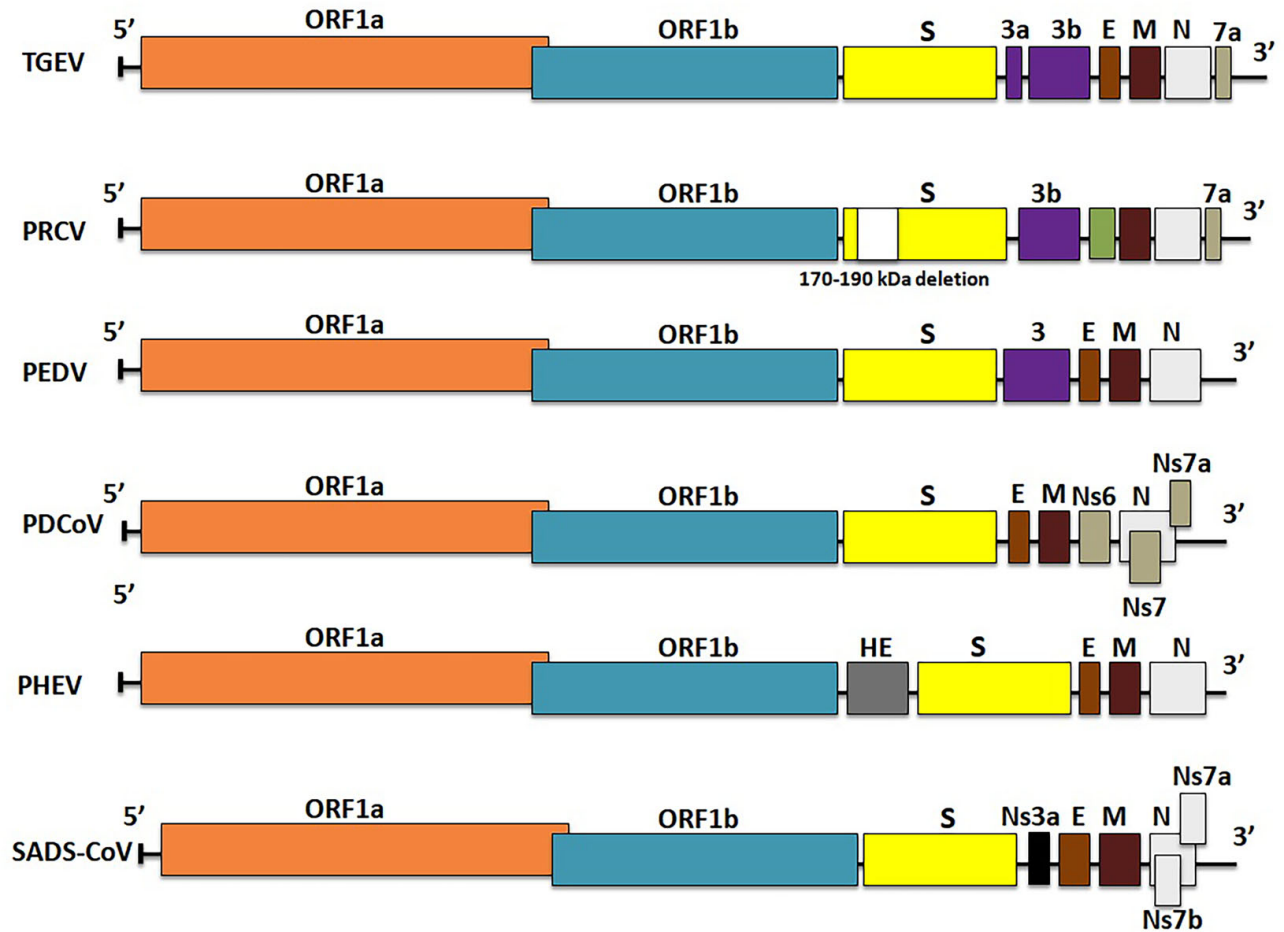
Schematic diagram of swine coronavirus (SCoV) virion (S, Spike S structural gene; E, envelope; M, membrane; N, nucleoprotein; HE, hemagglutinin-esterase; Ns3a, Ns6, Ns7, Ns7a, Ns7b are accessory genes) (Mora-Diaz et al. 2021).

Swine coronaviruses are single-stranded, polyadenylated, large genomic RNA (25–30 kDa) with positive sense polarity (. Similar genome profiles, replication strategies and protein expression are found in other animals’ and human CoVs (Chen et al. 2020). Swine coronaviruses are made up of four structural proteins: a large surface glycoprotein spike protein (S), an integral membrane glycoprotein (M), an envelope small membrane protein (E) and a nucleocapsid protein (N) (Song et al. 2019). Spike protein is a 180-kDa peplomer glycoprotein found on the virion envelope and the plasma membrane of infected cells. It contains epitopes for viral neutralization, diagnosis, and T-cell response to trigger immune response; and it is also required for virus cellular entry, as well as cell-to-cell fusion for some coronaviruses (Wang et al. 2020a). The spike gene contains factors of virus tropism and responsible for pathogenesis (Chen et al. 2020). Membrane (M) glycoprotein is a transmembrane protein with its carboxy-terminus embedded inside the virion core and it is thought to be essential in preserving the virion core structure (Kuo et al. 2016). The E protein is a membrane-associated polypeptide with a mass of 9.6 kDa that is essential for virus assembly (Castano-Rodriguez et al. 2018; Asrani et al. 2020). In addition, PHEV which is a beta-coronavirus contains hemagglutinin-esterase (HE) protein that forms second layer of shorter surface spikes (Turlewicz-Podbielska and Pomorska-Mól 2021). The nucleocapsid protein (N) with a mass of 60 kDa forms a helical structure of capsid inside the viral envelope by forming a complex with the genome RNA. The peplomers found in the envelope are shaped by spike protein trimers, giving the virions corona (crown-like) appearance (Wang et al. 2020a). The 3′-side of the genome encodes structural proteins such as E, N, S, M, HE and I 'internal protein' (encoded within the N gene) and HE (in certain beta-coronaviruses) as shown in Figure 3. The replicase locus is located at the 5′-end of the genome it is made up of two large open reading frames (ORFs), i.e. ORF1a and ORF1b, which together occupy about 20 kb of the genome. The replicase is translated into two polyproteins (pp) 1a and 1ab. These replicase polyproteins are further cleaved by the virus proteases into 16 proteins, most of which contains proteases, multiple RNA modification enzymes, a helicase, and a polymerase (V'kovski et al. 2021).

The TGEV genome is approximately 28.6 kb in size and consists of a 5′-end untranslated region (UTR), ORF1a/1b, S, M, E and N genes, and a 3′-UTR encoding three accessory genes such as 3a, 3 b, and 7 that are interspersed among the structural genes in the 3′-region (Chen et al. 2019). Cheng et al. (2020) conducted a study that revealed two distinct genotypes of TGEV, genotypes I and II, with genotype I further classified into subtypes Ia and Ib using maximum likelihood and Bayesian inference phylogenetic analyses. In comparison to TGEV, the PRCV genome has a significant deletion (621 to 681 nucleotides) near the N-terminus, resulting in a smaller S protein, and some variable region deletion in the downstream of S gene, impedes ORF3 expression (Saif and Jung 2020). Porcine epidemic diarrhea virus has a genome of about 28 kb that comprises four structural proteins (S, E, M and N proteins), ORF3 as an accessory protein and sixteen nonstructural proteins (nsp1-nsp16) (Vlasova et al. 2020). The SADS-CoV genome is approximately 27 kb in length and includes 9 ORFs: ORF1a, ORF1b, S, E, M, N genes and three accessory genes, NS3a, NS7a and NS7b (Zhou et al. 2018). Porcine delta coronavirus has a 25.4 kb genome that encodes 4 structural proteins [S, M, E and N], along with three accessory proteins (NS6, NS7 and NS7a) (Li et al. 2014; Wang et al. 2019). NS6 is anticipated to exist between M and N in the genome and it has recently been discovered as a virion-associated inhibitor of IFN-expression and has been proven to suppress the host innate immune response (), but NS7 is a 200-amino-acid peptide encoded by an alternate ORF within the N gene (Fang et al. 2017). Modulating viral pathogenicity, inducing apoptosis, and antagonising the IFN system are a few roles of these accessory proteins (Koonpaew et al. 2019). A previous phylogenetic analysis based on spike genes suggested that PDCoV was classified into three groups: China, the USA, and Southeast Asia (SEA). Southeast Asia PDCoV, is genetically distinct from China and the USA, but they share a common ancestor. The emergence and evolution of SEA PDCoV were explored using phylodynamic analysis methods based on complete genome. In the study, 18 complete genome sequences of SEA PDCoV identified between 2013 and 2016, as well as PDCoV from other regions, were analysed. The findings revealed that PDCoV was classified into two genogroups, G1 and G2. G1 is further subdivided into G1a (China), G1b, and G1c (USA). The G2 (SEA) group has further evolved into three clades: SEA-1 (Thailand), SEA-2 (Vietnam), and SEA-2r (Vietnam recombinant) (Stott et al. 2021).

### 3.1. Coronavirus replication

Coronavirus replication begins with interaction between the S protein and receptor for the viral entry into the host cell. Coronaviruses' host species range and tissue tropism are largely controlled by this interaction. The relatively variable S1 subunit mediates receptor binding, while the more conserved S2 subunit undergoes large conformational changes, resulting in virion and cell membrane fusion. The outer membrane, or in some cases, acidified endosomes are the sites of the membrane fusion (White and Whittaker 2016). Different CoVs in nature use diverse range of receptor to get entry inside the cell. Different SCoVs tropism and receptors are summarized in Table 1. In conjunction to receptor binding and fusion, coronavirus S proteins should be proteolytically cleaved by host cell-derived proteases (V'kovski et al. 2021). After genomic RNA uncoating and cytoplasmic entry, the RNA instantaneously translates the two large open reading frames, ORF1a and ORF1b into two polyproteins, pp1a and pp1ab. The specific non-structural proteins (nsps) that support the virus replication and transcription complex are generated from the polyproteins (pp1a and pp1ab) through cis- and trans-cleavage of the polyproteins by the virus proteases (V'kovski et al. 2021). After that, several of the nsps constructs to form replicase–transcriptase complex (RTC), that further develops environment to synthesize RNA, including transcription and replication of subgenomic RNAs (Wang et al. 2020a).

**Table 1.** Different swinecoronaviruses, tropism, and their receptors.

Genus	Virus species	Tropism	Receptor/Co-receptor	References
Alpha	TGEV	Respiratory and gastrointestinal system	Aminopeptidase N (APN), Sialic acid	Turlewicz-Podbielska and Pomorska-Mól (2021)
	PRCV	Respiratory system	Aminopeptidase N (APN)	Peng et al. (2020)
	PEDV	Gastrointestinal system	N acetyl neuraminic acid	Liu et al. (2015)
	SADS- CoV	Gastrointestinal system	Not identified	Li et al. (2018b)
Beta	PHEV	Respiratory, gastrointestinal and nervous systems	5-N-acetyl-9-O acetyl neuraminic acid (Neu5, 9Ac2), Neural cell adhesion molecule (NCAM)	Dong et al. (2015); Alluwaimi et al. (2020)
Delta	PDCoV	Gastrointestinal system	Aminopeptidase N (APN)	Wang et al. (2018)

The translation and assembly of viral replicase complexes are followed by synthesis of viral RNA including genomic and subgenomic RNAs. The order *Nidovirales* is having unique properties of nested set of transcription for production of subgenomic RNAs. Subgenomic RNAs work as mRNAs for the synthesis of structural and auxiliary/accessory genes. All positive sense subgenomic RNAs are from 3′ co-terminal which is independently transcribed, forming a set of nested RNAs. Both genomic and subgenomic RNAs are generated through negative-strand intermediates (Wang et al. 2020a). Each mRNA is translated to produce the protein product of its most 5′-gene, but it can also be translated to produce a second, downstream protein. It has a mysterious discontinuous transcription mechanism that allows them to replicate. Transcription regulating sequences (TRSs) at the 5′-terminal of each transcriptional unit are required to regulate subgenomic mRNA transcription (Du Toit 2020). The translation and incorporation of virus structural proteins S, E and M into the cell organelles such as endoplasmic reticulum occurs immediately after viral replication. These proteins find their path into the vesicular tubular cluster (VTC) through the secretory pathway (Wang et al. 2020a). However, viral genomes encapsidated by N protein bud into membranes of the VTC encompassing viral structural proteins, creating complete virions (V'kovski et al. 2021). The M protein guides most protein–protein interactions obligatory for assembly of CoVs. Subsequently, new virions bud from intracellular membranes and then excluded out from the cells through secretory vesicles by the process of exocytosis (Chen et al. 2020). Swine CoVs are considered difficult to propagate. However, certain studies like the use of embryonated chicken eggs have been utilized successfully to propagate deltacoronaviruses which can be helpful for vaccine production and isolation of coronaviruses (Iseki et al. 2021).

## 4. Coronavirus evolution

Novel viruses require access to new hosts, which is often achieved through ecological disruption and the ability to efficiently infect the hosts, which is frequently driven by adaptive evolution. Coronaviruses can quickly adapt to new hosts by using genetic recombination and mutation. A new virus cannot be created solely through point mutations but when the same host is infected with two coronavirus strains at the same time, leads to the recombination of hundreds or thousands of base pairs in long genomic fragments, resulting in the emergence of new virus (Trovato et al. 2020). Three new human and swine CoVs have emerged and expanded globally in the 21st century, highlighting the critical need of detection techniques for zoonotic coronaviruses (Wang et al. 2019). Mutation and recombination are the two major forces that drive coronavirus evolution. Coronaviruses have an increased frequency of homologous RNA recombination, but the mechanism is unclear. Evolutionary changes like mutation and recombination along with genetic drift and pleiotropy have a significant impact driving viral adaptations to various hosts. Phylogenetic studies have demonstrated evolutionary changes in the S glycoprotein of bovine coronavirus (BCoV) and human coronaviruses (hCoVs), and these modifications do not alter the viruses to retain in population, but it also influences the pathogenicity and the virulence (Miller 2005; Ren et al. 2006; Bidokhti et al. 2013; Lee and Lee 2018). Mutation in viruses induces the increased efficiency to bind with the host receptors, and some mutation occurs to evade the host immune response and assure continued dissemination by the host for successful transmission to another variety of animal (Oberemok et al. 2020). Viral cross-species transmission and infection rely on fast evolution, which is achieved through high genetic changes, recombination, along with the proximity of potential host species (Wu and Kewal Ramani 2006; Streicker et al. 2010; Lu et al. 2015; Abdullahi et al. 2020). Pathogenicity and dissemination capacity are increased by mutations that enable faster and efficient host entrance, maximize viral reproduction, and reduce sensitivity to host immune responses, either inside the host species or even across species (Longdon et al. 2014; Ahmed et al. 2020; Brook et al. 2020). Earlier cross-species transmission investigations have confirmed the necessity of monitoring genetic changes of virus in the new host. For example, in SARS-CoV-2, variations to the receptor-binding domain (RBD) modify virus infectivity, virulence and natural immunity or vaccination effectiveness (Korber et al. 2020).

Coronavirus (CoV) evolution is further controlled by genetic variation, viral mutation potential, and genetic recombination, along with surface glycoprotein adaptability, which is typically enhanced via host-parasite contact despite decreasing pathogenicity (Alexander and Day 2010; Alnazawi et al. 2017). On the other hand, polymorphisms in particular genes not just affect the gene's potential morphological expression but can also impact additional genetic traits and subsequent transcriptional activities of other genetic sequences (Bang 1978; Van Sluijs et al. 2017; Lee and Lee 2018). Coronavirus genome recombination can be aided by signals for RNA polymerase recognition. Recombination has only been confirmed between CoVs of the same genus, despite the possibility of intergroup recombination. Intragroup recombination has been reported between canine and feline enteric coronaviruses, mouse hepatitis virus (MHV) and infectious bronchitis virus (IBV) strains (Su et al. 2016). During coinfection, a polymerase-jumping system causes recombination among coronaviruses (Su et al. 2016). In cell culture, recombination between human (HCoV-OC43) and animal (bovine) CoVs has recently been reported. It has been proposed that members of group II CoVs like hemagglutinating encephalitis virus (HEV) have a high capacity for recombination (Wu et al. 2003). RNA viruses have been observed to mutate at frequencies ranging from 10^3^ to 10^5^ base substitutions per nucleotide (Duffy 2018). These values are higher than those seen during the replication of DNA virus and cellular DNA (Vlok et al. 2019). RNA viruses form into diverse populations consisting of ensembles of highly related but non-identical genomes referred as viral quasispecies as a result of their high mutational rate (Domingo and Perales 2019). The minimal copying fidelity shown by viral replicases is the molecular basis of all this uncertainty (Kautz and Forrester 2018).

Swine acute diarrhea syndrome (SADS) has recently been detected in piglets and it is caused by the SADS coronavirus (SADS-CoV), a new strain of coronavirus HKU2 from *Rhinolophus* bat (Zhou et al. 2018), but till now there has been no evidence of human infection. In the case of PDCoV, molecular studies indicate that interspecies transmission occurred recently as a result of contact between bird and mammal (Ma et al. 2016) and also virus progenitors were found among quails and sparrows (Li et al. 2018c). A recent report of finding porcine deltacoronavirus strains in three Haitian children’s serum samples with undiagnosed febrile illness also raises a question of interspecies transmission of other CoVs as till now humans get infected with only alphacoronaviruses and betacoronaviruses (Lednicky et al. 2021a). In fact, the broad cross reactivity of deltacoronaviruses with cellular receptor–aminopeptidase N (APN)–from different species also supports this kind of evolution (Vlasova et al. 2021).

The virus cannot evolve in isolation but is instead complemented by changes in the host as well. Host evolution and ecology, linked to genetic changes and recombination processes, offer the essential host variables that must occur concurrently with virus evolution (Fumagalli et al. 2010; Cui et al. 2013; Nasir et al. 2015; Lee and Lee 2018). Several variables must come along for successful zoonoses and the capacity to build in a new host, along with the development of a founder virus with a modification that enables higher levels of dissemination in the new host (Jelinek et al. 2021).

### 4.1. Recombination

Coronaviruses are experts at RNA recombination, a genetic process in which two separate viral genomes of RNA hybridize and swap RNA regions at specific locations. RNA recombination occurs frequently in the genomes of CoVs during replication and thereby integrity of RNA is maintained. Coronaviruses can also acquire new genes, which allows them to accommodate to a new host. Variations in other regions of the CoV genome can either make it more adaptable to the new host, or the genome can incorporate host/cellular RNA that codes for an accessory component that enables it to replicate in a different species. As a result, it is clearer than ever that new CoVs pose a significant risk (Alluwaimi et al. 2020). RNA recombination events that result in mutations of S protein are classic explanations of how viral genomic modifications enable a coronavirus to evolve and bind to a target ligand, allowing it to infect a new species (Ji et al. 2020).

Due to the high degree of mutation that defines their coexistence in a diverse range of variants, CoVs appear as quasispecies (Mandary et al. 2019). CoV RNA mutation rates have been reported to be moderate to high. The estimated annual substitution rate for CoVs was 10^4^ substitutions per site. For example, the IBV hypervariable area of the S gene's nucleotide mutation level was determined to be 0.3–0.6 × 10^2^ per site annually. In 229E CoV, the rate of substitution was determined to be ∼ 3 × 10^4^ per site per year, while in SARS-CoV, the rate was determined to be 0.8–2.38 × 10^3^ nucleotides per site annually (Su et al. 2016). As shown during the SARS outbreaks in 2002–2003, the phylogeny and biology of the coronavirus (CoV) was likely characterized by frequent host-shifting events, whether animal-to-human (zoonosis) or human-to-animal (reverse zoonosis) (Lau et al. 2018). SARS-CoV has shown evidence of recombination with PEDV at some point of evolutionary history (Su et al. 2016).

### 4.2. Interspecies transmission

**Figure 4.**
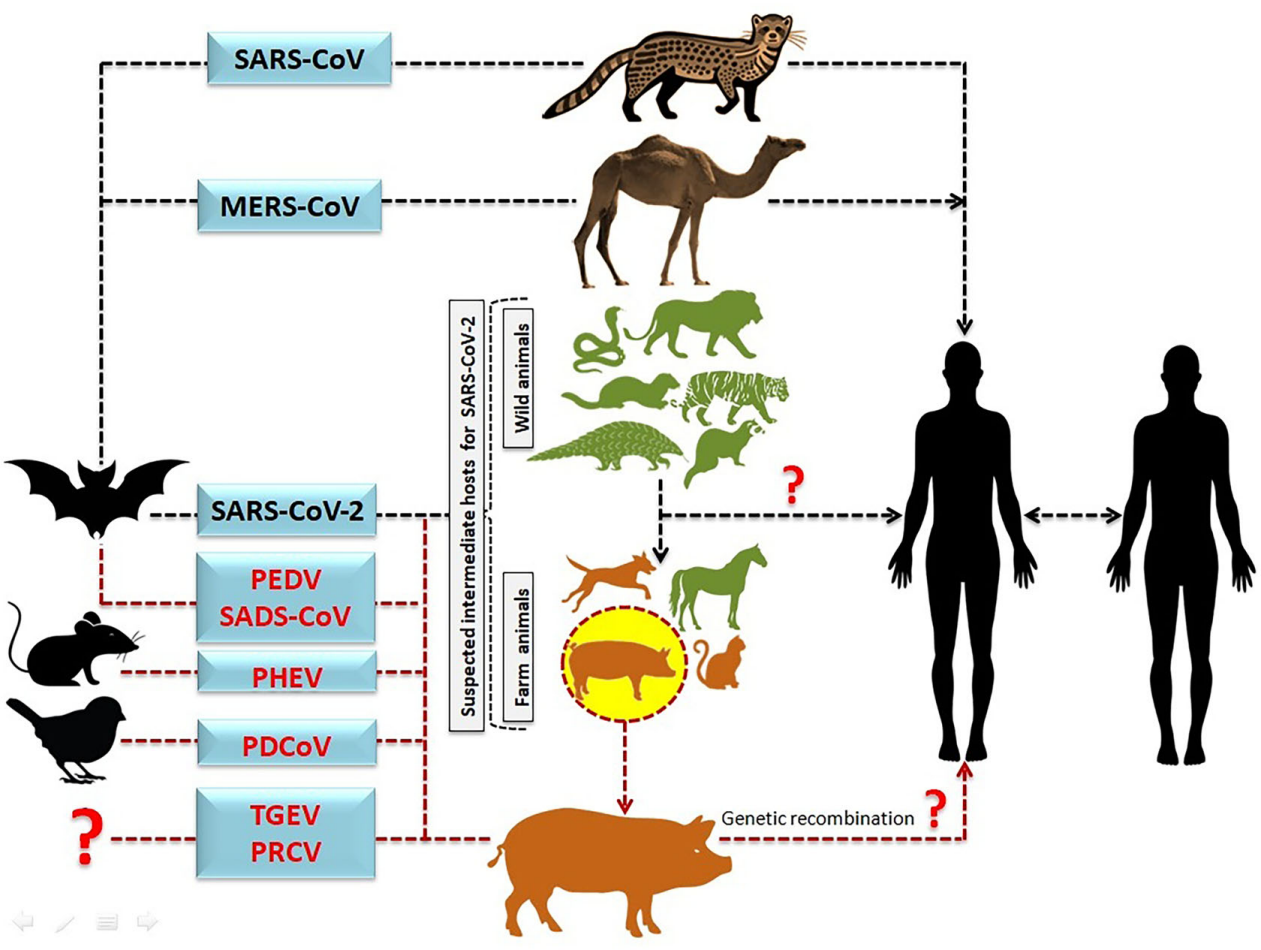
Diagrammatic depiction of the source of SARS-CoV-2 from bats and presumed intermediate hosts, such as farm and wild animals. (The transmission of MERS-CoV and SARS-CoV to humans *via* an intermediate host camel and palm civet cat, respectively. It also illustrates the likelihood of SARS-CoV-2 originating from pigs after genetic recombination, which would enable the SARS-CoV-2 to cross the species boundary and transmit disease to humans.)

The definition ‘Species barrier’ refers to natural mechanisms that prevent pathogens from spreading from one species to another. Spill-over is the transition of an organism from a host to another recipient species, as shown in Figure 4. Pathogens can propagate in a variety of ways, including from severely diseased domestic animal hosts to non-domestic hosts (Borremans et al. 2019). A complex pathogen transmission is being created by these pathogens spill-over and emergence events. Apart from the dietary and cultural factors, the ecological, epidemiological and human inherent factors may also link with spill-over of food-borne zoonotic diseases (Dhama et al. 2013a; Dhama et al., 2013b; Dhama et al., 2013c; Iturriza-Gomara and O'Brien 2016; Plowright et al. 2017). The interaction of viral-specific S protein, with the host ACE2 receptor supports spillover of CoV (Salata et al. 2019; ; Tiwari et al. 2020). Mutations in receptor binding domain (RBD) of S protein cause changes in receptor specificity, interaction and binding affinity, resulting in changing of transmissibility, pathogenicity, and cross-species jumping, as well as a predisposition to latest and more catastrophic diseases (; Wang et al. 2020c). Before 2002, research showed that mammalian CoVs may infect birds and cross-species barriers. Even though poultry are vulnerable, chicks and pigs were not susceptible to bovine coronavirus infection (Ismail et al. 2001; Saif and Jung 2020).

Experiments have shown that numerous subgroups of CoVs, such as canine enteric CoV (CECoV) and feline CoV (FCoV), are strongly linked to the TGEV/PRCV cluster, with interspecies transmission. All of these viruses are closely related biologically, antigenically, and genetically, and they could be host range variant of a CoV (Saade et al. 2020). They cross-react with monoclonal antibodies (mAbs) targeting to the S, N, or M protein in virus neutralization tests. They all share identical antigenic sub-site A on the S protein. Interestingly, antibody-dependent enhancement (ADE) of FCoV (feline infectious peritonitis virus [FIPV] strains) infection on macrophages cell culture is efficiently facilitated by neutralizing immunoglobulin G (IgG) mAbs against S protein of TGEV, illustrating the functional cross-reactivity of the S antibodies. The N and M proteins of type II FCoV, CECoV, and TGEV share >90% identical amino acids and the S protein shares >80% lengthwise identical amino acids (Saif and Jung 2020). Aminopeptidase N is a cellular receptor shared by TGEV, PRCV, and FCoV. The cross infection can occur between pigs, dogs and cats, causing symptoms such as profound blood-spattered diarrhea of which its occurrence remains mysterious (Saif and Sestak 2006). Amino acid composition of APN receptor in human, feline, and swine CoVs show 78% identity. The APN receptor used by alpha CoVs, on the other hand, is species-specific, and the feline APN is a functional receptor for other apha CoVs, including TGEV. Because of the characteristics of feline APN, cats can get infected with TGEV with or without symptoms (Parkhe and Verma 2021). A chimeric virus with a PEDV spike protein and TGEV backbone was identified as a variant genotype of TGEV strain with unique deletions and distinct amino acid changes similar to PRCV, implying recombination between the variant TGEV, PEDV, and PRCV (Belsham et al. 2016; Boniotti et al. 2016). Lednicky et al. (2021b) discovered porcine deltacoronavirus strains in plasma samples from three Haitian children suffering from acute undifferentiated febrile illness. Human infections were caused by at least two distinct zoonoses with the same mutational signature in the genes encoding Nsp15 and the spike glycoprotein, according to genomic and evolutionary analyses. Bat CoVs have a broad range as well as a great potential for spill-over in multiple animal species. As previously reported in the civet cat and dromedary camel, which culminated in the well-known catastrophes SARS and MERS, respectively, whereas SADS is latest spill-over in pigs which showing 95% genome similarity with bat CoV and causing high mortality (24,693 death) in neonatal piglets (Zhou et al. 2018).

## 5. Porcine CoVs: pathogenesis and clinical signs

### 5.1. Transmissible gastroenteritis virus (TGEV)

The most frequent clinical manifestation in piglets is vomiting, accompanied by milky yellow diarrhea, dehydration, and weight loss. Death may occur in 2–7 days after the onset of illness in animals less than the age of 7 days. In-appetence and diarrhea last for a shorter period in older pigs, but subclinical infections can often be seen. Extreme symptoms in lactating sows include pyrexia, vomiting, diarrhea, and failure of sow to produce milk. The time-period for the disease to develop can last up from 18 hours to 3 days (Mostl 1990). Xia et al. (2018) reported endemic form generally lasts for short period of time, but in susceptible animals, it may remain in an enzootic form most of the time. The majority of sows in these herds become infected and their suckling piglets develop passive immunity. In such circumstances, diarrhea is more likely to occur after weaning (Zhang et al. 2017; Xia et al. 2018). Gross pathology is limited to the gastrointestinal tract that includes a bloated stomach with fluid-filled intestine which having undigested milk, gas and revealed flabby consistency on palpation. Damage to the villous enterocytes causes thinning of the intestinal wall which can be seen when segments of the intestine are immersed in an isotonic buffer and examined underneath a dissecting microscope. The villous atrophy is marked in jejunum and to some extent in ileum but not in proximal duodenum (Guo et al. 2020). Xia et al. (2018) observed that newborn pigs had severe villous atrophy compared to 3-week-old pigs, implying that neonates are more prone to more severe TGEV infection. The functions of villi are quickly restored after recovery since the intestinal crypt epithelial cells continue to regenerate (Fenner 2017). The mammary gland tissues and alveolar macrophages have been identified as extra-intestinal sites for replication of TGEV (Vlasova et al. 2020). Transmissible gastroenteritis virus induced pyroptosis in porcine intestinal cell line and produce pro-IL-1*β* by caspase-1 activation (Wei et al. 2021). Porcine kidley-15 (PK-15) cell lines are preferred to isolate TGEV causing cytopathic effect (CPR)- like cell fusion, cell rounding, shrinkage, and cell detachment from the surface of the culture vessel (Yuan et al. 2021).

### 5.2. Porcine respiratory coronavirus (PRCV)

Patho-morphological study of PRCV revealed macroscopic and microscopic lesions which are typically confined to the lungs and manifest as lung consolidation, bronchointerstitial pneumonia with recurrent peribronchiolar and perivascular lymphohistiocytic cuffing (Cui et al. 2020). The similarity of nucleotide and amino acid sequences between TGEV and PRCV genomes is 96–98%, implying that PRCV originated from TGEV. Two aspects of the PRCV genome that may enlighten its altered tissue tropism which are a significant exclusion (621–681 nt) in the 5′ of the S gene, resulting in a smaller S protein, and in constant sequence alterations of ORF3 gene (Saif and Jung 2020).

Porcine respiratory coronavirus has a tropism for both the upper and lower airway passage similar to SARS-CoV and SARS-CoV-2. Similar to PRCV in pigs, most individuals infected by SARS-CoV-2 or SARS-CoV has an asymptomatic or subclinical infection and recuperates. Respiratory disease can be persistent in seriously affected patients, and it can be exacerbated by a storm of cytokines and multi-organ failure. In a non-human primate model, SARS-CoV-2 and SARS-CoV caused extreme diffuse alveolar damage and pulmonary edema due to excessive degeneration of type 1 pneumocytes of lung alveoli (Rockx et al. 2020). For PRCV diagnosis and differentiation from TGEV, real-time reverse transcription polymerase chain reaction (qRT-PCR) was used (Saif and Jung 2020). Using swine testicle and pig kidney (PK) cell lines, PRCV was isolated from nasal swab fluids or lung tissue homogenates and propagated in cell culture (Saif et al. 2019). Porcine respiratory coronavirus serology is complicated owing to cross-reactivity with TGEV.

### 5.3. Porcine epidemic diarrhea virus (PEDV)

The PEDV pathogenesis is more related to that of TGEV. The destruction of epithelial cells in the small and large intestine causes villous shortening. Viral replication takes place in the cytoplasm of epithelial cells of villi over the small intestine within 12–18 hours of post infection (PI) and a maximum effect was reached at 24–36 hours. Enterocyte degeneration occurs as a result of the infection, lowering the ratio of intestinal villous to crypt from 7:1 to 3:1 (Jung et al. 2020). Clinical manifestation of diarrhea, vomiting along with dehydration occurs after a 22–48-hour incubation period and is difficult to distinguish from TGEV symptoms. Unlike TGEV, the most affected groups are feeder and weaned pigs (Wu et al. 2020). In suckling piglets, PEDV can cause serious clinical disease and mortality may reach approximately to 80–100%. TGEV propagate more rapidly in diseased herds, resulting in a gratifying serious syndrome, but Weng et al. (2016) indicated that TGEV cases in the previous year have shown an altered clinical pattern, resembling PEDV. Porcine epidemic diarrhea virus and TGEV have very close pathogenetic mechanisms that result in the same immunological condition. Recent studies also showed increased PEDV pathogenicity on co-infection with porcine delta-coronavirus (Zhang et al. 2021c). A study also showed the RBCs as a possible vehicle for transmission of PEDV which could also be possible in other coronaviruses (Li et al. 2021). Immunity to PEDV is dependent mostly on secretory antibody in mucosa of small intestine, which is only present for a brief period of time following disease. Antibody transfer through milk protects suckling piglets, but circulatory antibodies do not. To diagnose PEDV, blocking-ELISA is mainly used to detect viral antigen or by an immunofluorescence assay (Magtoto et al. 2019). El-Tholoth et al. (2021) designed a new, low-cost, rapid, semi-quantitative, field deployable, 3 D-printed microfluidic device for auto-distribution of samples and self-sealing, as well as real-time and reverse transcription-loop-mediated isothermal amplification (RT-LAMP), allowing the co-identification of PEDV, TGEV, and PDCoV in around 30 minutes.

### 5.4. Porcine hemagglutinating encephalomyelitis virus (PHEV), vomiting and wasting disease (VWD)

The virus would be particularly transmitted through nasal secretions. In pigs, the respiratory tract is the primary site for PHEV replication. The virus replication occurs in the interior of the upper respiratory tract epithelium without inflicting clinical symptoms in majority of cases. Afterwards the virus moves towards the brain and spinal cord through peripheral nerves, resulting in encephalomyelitis or VWD. Several pathways were used by the virus to disseminate from the initial replication sites to the CNS via peripheral nervous system. One pathway connected the nasal mucosa and tonsils to the trigeminal ganglion and trigeminal sensory nucleus in the brain stem. The vagal sensory ganglion provided a second route from the vagal nerves to the vagal sensory nucleus in the brain stem. Following replication in local sensory ganglia, a third pathway reaches the spinal cord from the intestinal plexi (Mora-Diaz et al. 2019). Almost all piglets under the age of three weeks show severe clinical signs. They vomit frequently, pale, listless and huddle together after a 4–7 day-incubation period. Mora-Diaz et al. (2021) proposed that vomiting is due to virus replication within infected neurons or through vagal sensory ganglion in the vomiting center. Widespread muscle twitching and hyperesthesia are typical symptoms of encephalomyelitis but opisthotonus, blindness and nystagmus may also occur. Weakness is followed by syncope and collapse in the large number of piglets. An interruption in stomach emptying was thought to be the cause of the wasting (Mora-Diaz et al. 2019).

Even though PHEV is found in pig populations all over the world, clinical disease is rare. Due to the fact that the majority of pigs are seropositive, their young ones are shielded by maternal antibodies until they acquire age-dependent protection. Asymptomatic infections develop in pigs between ages of 8 and 16 weeks throughout the passive immunity phase, inducing active immunity (Mora-Diaz et al. 2021). Cachexia, stomach dilatation and abdominal distension have been reported as the only gross lesions in some chronically infected pigs with natural PHEV infections (Mora-Diaz et al. 2020). Histopathological lesions such as epithelial degeneration and infiltration of inflammatory cells are visible in the tonsils and respiratory tracts of severely diseased pigs. Non-suppurative encephalomyelitis was reported in 70–100% of the pigs with nervous symptoms and 20–60% of the pigs with VWD syndrome. Gliosis, perivascular cuffing and neuronal degeneration are the characteristic lesions (Mora-Diaz et al. 2019). Porcine hemagglutinating encephalitis virus is similarly interrelated to bovine, canine, murine, equine and human CoVs, accompanied by rat sialolodacryoadenitis coronaviruses (Mora-Diaz et al. 2019). Porcine hemagglutinating encephalitis virus has an envelope-associated glycoprotein layer (hemagglutinin-esterase) that allows it to hemagglutinate and hemadsorb chicken, mouse, hamster, rat, and turkey erythrocytes, that distinguishes it from other swine coronaviruses (Sasseville et al. 2002; Mora-Diaz et al. 2021).

### 5.5. Swine acute diarrhea syndrome coronavirus (SADS-CoV)

In Guangdong province, China, the swine enteric *Alphacoronavirus* (SeACoV) was identified in 2017. It is also known as SADS-CoV and porcine enteric *Alphacoronavirus* (PEAV). Initially, epidemics of profound diarrhea in nursing piglets were reported at four pig farms in a hilly region of north Guangdong (Gong et al. 2017; Zhou et al. 2018). Swine acute diarrhea syndrome coronavirus was first recovered in the fecal material of young pigs with severe diarrhea as well as an increase in fatality rate (Pan et al. 2017). Virus resistance and species tropism were also studied in cell lines derived from tissues of humans and several animal species, including bats, mice, rats, gerbils, hamsters, chickens, pigs, and nonhuman primates.

Swine acute diarrhea syndrome coronavirus, like other pigs enteropathogenic viruses, is spread via the fecal-oral route. This was shown in pigs using cell-cultured SADS-CoV strains during challenge trials (Pan et al. 2017; Zhou et al. 2018; Xu et al. 2019). Swine acute diarrhea syndrome coronavirus is most likely spread through vomitus or diarrheic stool from diseased piglets. Other PCoVs may be capable of infecting pigs in a number of ways. For instance, PEDV can cause intestinal mucosal infection if it enters in the body through the nose (Li et al. 2018b). Future studies should also focus on the infectious nature of aerosolized SeACoV.

SADS-CoV could produce a similar public health risk if it is transmitted from pigs or other intermediate hosts to human (Yang et al. 2019). Based on recent molecular studies, SADS-CoV has been discovered to have the potential for further cross-species transmission, including the ability to infect primary human airway, fibroblasts, human nasal epithelial, microvascular endothelial cells (MVE), and intestinal cells (Goldstein et al. 2021). *In vitro* experiments showed that SADS-CoV can grow effectively in monkey and nine human cell lines, suggesting that it has zoonotic potential. Since a conserved receptor for SADS-CoV cell entry has been proposed across various animal species, *via* interspecies transmission from porcine, SADS-CoV may pose a potential threat to human health in the future. Similar to other porcine enteric CoVs, SADS-CoV infects intestinal epithelial cells as its primary target cells (Koonpaew et al. 2019; Wang et al. 2019). Studies reported that in the intestine of SADS-CoV infected piglets proclaimed significant atrophy of villi causing devastating pathological lesions (Zhou et al. 2018; Koonpaew et al. 2019; Wang et al. 2019; Xu et al. 2019). The pathophysiology of SADS-CoV and the natural resistance to it are either poorly understood or conflicting. More investigations into the pathogenesis of this disease are required.

### 5.6. Porcine deltacoronavirus (PDCoV)

Although the etymology of PDCoV in USA swine is unidentified, serologic data show that it was propagating in swine prior to its discovery in 2014 (Sinha et al. 2015). Porcine *Deltacoronavirus* has also been identified in Canada (March 2014), Korea (April 2014), Thailand (2015), mainland China (2015), Vietnam and the Lao PDR. According to a study in Korea, only two samples from one farm were PDCoV RNA positive out of 691 diarrheic pig fecal samples collected from 2014 to 2015 (Lee et al. 2016). The SL2 and SL5 Korean PDCoV strains were genetically related to the USA PDCoV strains, whereas they were not related to the older Korean strain, KNU14-04. There was a high occurrence of PDCoV (>30%) and periodic co-infections with PEDV (51%) reported. The global PDCoV strains and Chinese PDCoV strains shared a high nucleotide identity which is around 98.9%. The Thai PDCoV strains had the highest nucleotide identity (98.4%) with the Chinese strain CHN-AH-2004 (Janetanakit et al. 2016), but they formed a different cluster from the Chinese and USA strains (Zhang 2016). Strains of the Thai PDCoV lineage were detected in Lao PDR, while strains of the USA PDCoV lineage were identified in Vietnam (Saeng-Chuto et al. 2017). Recent studies have established four distinct phylogenetic lineages of PDCoV (USA, China, Thailand, and Early China lineages); each with its geographic distribution pattern (He et al. 2020). In piglets, PDCoV causes diarrhea, vomition, dehydration with typical histopathological lesions of atrophic enteritis (Zhang et al). The fecal-oral route is the most common way for PDCoV transmission. The infection is spread mostly by feces, blood, and other infected fomites. The duration of experimentally produced PDCoV diarrhea was 5–10 days, and fecal viral shedding persists upto 19 days (Hu et al. 2016). After recovering from an infection, pigs mostly tend to shed PDCoV in their feces. Hence, subclinically infected or convalescent carriers may be another potential reservoir for PDCoV.

Porcine *Deltacoronavirus* is having hemagglutinating (HA) property which has been documented by Zhang et al. . The virus had no HA reactivity for the pig, guinea pig, mouse, chicken or human erythrocytes, but it can cause agglutination of rabbit red blood cells when pre-treated with neuraminidase or trypsin. Furthermore, the results of the HA assay exhibited a positive relationship with the infectious viral titer. The findings show that assessing PDCoV HA activity may be a promising diagnostic tool for inspecting and monitoring PDCoV disease in pig population (Zhang et al).

## 6. Could pig be a vector for SARS-CoV-2?

Pigs are vulnerable to a variety of CoVs, including TGEV, PEDV, PRCV and the newly discovered SADS-CoV (Pan et al. 2017; Zhou et al. 2018). These viruses seem to have emerged in bats, similar to SARS-CoV and MERS-CoV. Pigs are also vulnerable to a *Betacoronavirus*, PHEV (Mora-Diaz et al. 2019), *Deltacoronavirus* CoV, and PDCoV (Zhang et al). As stated earlier, pigs and humans have distinct ranges of CoVs that do not overlap significantly. It is crucial to highlight that CoVs primarily affects the respiratory system in humans and enteric system in pigs. Coronaviruses have a very high mutation rate since they are RNA viruses, which help them to produce new strains/variants and adapt to a wide variety range of hosts. As a result, all identified CoVs of human have animal origins, according to genome sequences (Cui et al. 2019). In pigs, only one CoV is related to respiratory infections (PRCV), which causes mild disease and lesions in majority of cases (Cui et al. 2020) and is thus of little interest to the farmers. Only a few studies have looked at whether SARS-CoV or MERS-CoV can infect pigs, and whether pig viruses can infect humans. RNA of SARS-CoV was found in one pig during a survey of 6 domestic animal species, which also includes pigs, and 242 individual pigs in China (Opriessnig and Huang 2020). Also, two of the 242 pigs tested antibody positive for SARS-CoV. According to the researchers, interspecies transmission of SARS-CoV is possible and could pose a threat to human population (Opriessnig and Huang 2020).

Moreover, SARS-CoV replication was also supported by successive cultures of virus in turbinate cells of pigs (PT-K75) (Weingartl et al. 2004). MERS-CoV has also been shown to be amplified in pigs. Currently, there is no proof that pigs can be diagnosed with SARS-CoV-2 or they can spread the virus. Since the SARS-CoV-2 infection is still underway, all testing at this point focuses on detecting affected individuals to avoid more human-to-human transmissions. Early pandemic warnings were focused on pigs because they have been known to incubate other viruses, such as influenza, and they live in large numbers in close vicinity to humans in China, the place where the pandemic began, where 300 million pigs were farmed (Mallapaty 2021). SARS-CoV-2 replication was found to be successful in cats and ferrets but ineffective in pigs, rats, chickens, and ducks in an experimental infection study (Shi et al. 2020).

Pigs are generally not found susceptible to SARS-CoV-2 and may tend to have a low susceptibility to viral infection, requiring a very high infectious dosage to elicit a low degree of infection (Schlottau et al. 2020; Younes et al. 2021). In one experimental study, SARS-CoV-2-inoculated domestic pigs showed no evidence of clinical signs, virus replication or virus-specific antibody responses, thus were not found susceptible to the viral infection on oral/intranasal/intratracheal challenge, revealing that pigs are unlikely to be potential carriers of SARS-CoV-2 and neither to be considered a suitable animal model for SARS-CoV-2 (Meekins et al. 2020). Certain pig breeds like Ossabaw pig can be developed as obese and could present nearly all features of the metabolic syndrome, thus resembling to more than 80% of the critically ill COVID-19 patients, hence infection with porcine respiratory coronavirus of metabolic syndrome pigs (obese Ossabaw pig) has been suggested to considered as a highly relevant animal model of severe COVID-19 (Heegaard et al. 2020).

Pigs are most likely to contract SARS-CoV-2 because they have the same ACE2 receptor binding sites as humans (Wan et al. 2020). Several lines of evidence suggest that pigs could be prone to SARS-CoV-2 infection, for both natural and laboratory environments, and exhibit seroconversion. SARS-CoV-2 can use the ACE2 receptor from animal species, including porcine ACE2, to enter the cell *in vitro*, implying that pigs may be vulnerable to the infection of SARS-CoV-2 (Zhou et al. 2020b). Evaluating ACE2 receptor-mediated SARS-CoV-2 entry across different animal species, a key determinant for determining the range of hosts that could be infected by this pandemic virus, it was observed that ACE2 of *Sus scrofa* (wild pig) facilitated virus entry into non-susceptible cells (Zhang et al. 2021c). Receptor binding domain of spike protein of SARS-CoV-2 links with the pig ACE2 entry receptor with comparable efficiency to ACE2 entry receptor of human, as per structure-based observations (Wan et al. 2020). Pigs will co-express entry receptor ACE2 and TMPRSS2 protease as a viral activating factor in different cell lines, according to single-cell screening, and SARS-CoV-2 replicates in different cell lines of pigs (Chu et al. 2020; Schlottau et al. 2020; Zhou et al. 2020b). SARS-CoV-2 was able to replicate in two different porcine cell lines, *viz*., swine testicle (ST) and porcine kidney (PK-15) cell lines that were found permissive for the virus, inducing cytopathic effects (Meekins et al. 2020).

Based on current understanding, the risk of contracting SARS-CoV-2 during a transplant operation involving pig-derived products should be very low, although it should be extensively investigated. Donor pigs should be tested for viral RNA of SARS-CoV-2 by PCR or antibodies by serology to rule out any infection. This protocol for human blood donors is being used in Wuhan and Hubei provinces, China (Chang et al. 2020). Various studies have revealed that different kinds of foods and food products including fruits, vegetables, bread, dairy products, and ready-to-eat foods, meat and meat products may act as potential vehicles/carriers for transmission of SARS-CoV-2 *via* carry-through or carry-over contaminations, therefore adequate precautious are warranted during handling of foods and in food processing units especially related to meat industry (Faslu Rahman et al. 2020a, 2020b; Hedman et al. 2021; Hu et al. 2021; Jo et al. 2021; Mallapaty 2021; Yekta et al. 2021). Hence, conclusive evidence of pigs getting SARS-CoV-2 infection is limited with some studies validating in favor and some against it, therefore explorative studies and larger monitoring investigations are required to conclude towards the role of pigs in SARS-CoV-2 transmisison and spill-over events to humans during the ongoing pandemic (Damas et al. 2020; Meekins et al. 2020 Zhao et al. 2020; Ma and Gong 2021; Pickering et al. 2021).

## 7. Conclusion and future prospects

Coronaviruses mostly affect the respiratory and intestinal tracts of both humans and animals. The potential of CoVs to mutate and accommodate to a different new environment renders them a constant threat to humans and animals, particularly in the swine industry workers, veterinarians, and animal handlers. Porcine CoVs, such as TGEV, PRCV, PEDV, PHEV, PDCoV, and SADS-CoV, are more common in piglets than in adults, so a strategic plan is needed to maintain a maternal level of antibody in newborns so that mortality can be reduced. Unified identification methods for TGEV, PRCV, PEDV, PHEV, PDCoV and SADS-CoV, including serological and virological methods, are required to prevent potential outbreaks. Past outbreaks of SARS-CoV and MERS-CoV, along with recent SARS-CoV-2 outbreaks have shown that the virus will cross species barriers and affect people, necessitating close monitoring and prompt updates to avoid potential losses. Since pigs are infected with a variety of CoVs, it is possible that SARS-CoV-2 may also be from pigs, as a result of genetic variation. SARS-CoV-2 infections in animals are becoming more common, highlighting the need to better understand and assess animal vulnerability, which is important for public health and the economy. Because only a few studies have looked into whether pigs can be infected with SARS-CoV, MERS-CoV, and SARS CoV-2, more research is needed to combat future pandemics in pigs and humans.

The recent and rapid global spread of highly pathogenic variants of SADS-CoV, PEDV, and PDCoV highlights the serious One Health threat posed by a newly emerged swine coronavirus, and that highlights the need for resources to better understand the virus and its disease-causing capability in mammals. Researchers must investigate the zoonotic potential of all PCoVs, as well as emerging porcine CoVs like PDCoV and SADS-CoV, so as to avoid future pandemics. Systematic monitoring and tracking of the proliferation of novel variant viruses in human and animal species can be done using the One Health approach. Intensive animal monitoring and risk assessments, particularly during and after an outbreak, will lead to an understanding of the diseases in natural animal reservoirs and to prevent spillover events. Swine farm in rural areas frequently utilize leftover foods from city restaurants as hogwash, without thoroughly fermenting it. Since there is no direct evidence of human-to-swine SARS-CoV-2 transmission, a legal notification must be issued to avoid such a practice, or legislative procedures should be implemented to prevent disease transmission through this route. The rising incidence of novel human and animal CoVs prompts the development of new methods for identifying higher-risk strains that can prevent forthcoming disease outbreaks. The majority of newly emerging human and animal CoVs, including SARS-CoV-2, is thought to have originated in bats and have the ability to adapt and propagate rapidly throughout the world. With increased global travel and frequent human-to-human interaction, it's more important than ever to develop and implement strategies for assessing the potential risk of emerging viruses to One Health globally.
